# The Cost of Screening Blood Donations for West Nile Virus

**DOI:** 10.1371/journal.pmed.0030062

**Published:** 2006-01-24

West Nile virus (WNV) was first isolated in 1937 from a sick woman living in the West Nile District of Uganda. Since then, it has been found in other parts of Africa, Europe, the Middle East, and central Asia, as well as around the Pacific. Then in 1999, it arrived in the Queens borough of New York City. During this first season, it caused 62 cases of encephalitis and seven deaths. WNV has since then spread across the United States, and can now be found in most states.

WNV is a flavivirus, a type of RNA virus. Like many other flaviviruses, including the dengue and yellow fever viruses, WNV is a blood-borne virus that is passed to people through mosquito bites; in the case of WNV, the mosquitoes acquire the virus predominantly by biting infected birds. Most people infected with WNV have no symptoms, but 20% of them develop West Nile fever, a flu-like disease. About 1% of infected individuals—usually older people or those with a weak immune system—develop severe neuroinvasive disease, either encephalitis or meningitis, which can cause long-term health problems or death. In 2005, about 100 people in the US died after infection with WNV.

Virtually all WNV transmission is through mosquito bites, but a few cases of WNV infection, some of them fatal, have been linked to contaminated blood transfusions. Consequently, in 2004, the US Food and Drug Administration (FDA) mandated that blood donations must be screened for WNV. The FDA did not recommend a specific screening method. In an ideal world, screening would aim to reduce the risk of contracting WNV from a blood transfusion as much as possible, at any cost. In the real world, however, the health benefits of any screening methodology (lives saved and improvements in the quality of life) have to be balanced against the costs of screening. To find out where this balance lies for WNV, Caroline Korves, Sue Goldie, and Megan Murray have estimated the cost-effectiveness of different strategies for screening blood donations for WNV and now report their results. [Fig pmed-0030062-g001]

**Figure pmed-0030062-g001:**
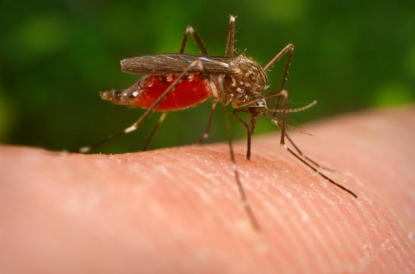
A number of mosquito species can transmit WNV, including Aedes japonicus, depicted here

The researchers used a computer-based mathematical model to compare different screening strategies. The baseline strategy was a donor questionnaire—blood donors reporting a recent fever cannot donate blood. The other strategies tested were nucleic acid testing of pools or individual samples of blood for WNV, universal screening versus screening restricted to donations destined for immunocompromised recipients, and seasonal screening versus screening throughout the year (WNV transmission peaks in late summer/early fall). The researchers modeled the cost-effectiveness of these strategies in areas with high levels of WNV transmission over a long season (as occurred in Mississippi in 2002), high transmission over a short season (Nebraska 2002), and low transmission over a short season (Massachusetts 2002).

Korves and colleagues found that in low-transmission areas with a short season, screening by questionnaire alone was the most cost-effective strategy—any other strategy was unlikely to prevent any cases of serious illness despite greatly increasing costs. In areas with high transmission, the best approach was seasonal screening by nucleic acid testing of individual donations earmarked for immunocompromised recipients. The researchers also discovered that seasonal screening of all donations provided little additional clinical benefit and was prohibitively expensive, and that screening throughout the year provided no additional benefit in any setting.

These results indicate that the currently mandated policy of screening donated blood for WNV may not be the best public health strategy. More restricted screening strategies may be preferable, suggest the investigators, with individual states adopting screening strategies that reflect the intensity and duration of their West Nile epidemics. The researchers also note that their estimates of the cost of WNV blood screening strategies were greater than generally accepted cost-effectiveness thresholds for health interventions. Thus, the resources spent in preventing rare cases of WNV infection arising from blood transfusion might be better used to reduce WNV transmission through controlling mosquito vectors. If such an approach were successful, they suggest, it might obviate the need for screening blood for WNV in many areas.

